# Öffentliche Gesundheit multidisziplinär fördern: Integration von Behavioral and Cultural Insights in den Public Health Action Cycle

**DOI:** 10.1007/s00103-025-04102-9

**Published:** 2025-07-21

**Authors:** Benjamin Ewert

**Affiliations:** Fachbereich Gesundheitswissenschaften, Hochschule Fulda, Leipziger Straße 123, 36037 Fulda, Deutschland

**Keywords:** Behavioral Public Policy, Public Health, Verhältnisprävention, Verhaltensprävention, Adipositas, Behavioral Public Policy, Public health, Structural prevention, Behavioral prevention, Obesity

## Abstract

Der Einsatz von Behavioral and Cultural Insights (BCI) im Bereich Public Health zielt darauf ab, die Gesundheit der Bevölkerung durch einen multidisziplinären und multimethodologischen Ansatz zu fördern. BCI integriert Erkenntnisse aus Verhaltensökonomie, Psychologie sowie Sozial- und Kulturwissenschaften, um sowohl individuelles Verhalten als auch strukturelle Rahmenbedingungen zu adressieren. Die Integration von BCI in den Public Health Action Cycle (PHAC) ermöglicht es, in jeder Phase – von der Problembestimmung über die Strategieformulierung und Umsetzung bis hin zur Evaluation – verhaltens-, sozial- und kulturwissenschaftliche Faktoren zu berücksichtigen.

Ein um BCI erweiterter PHAC beinhaltet die Planung und Umsetzung von effektiveren Public-Health-Interventionen, die sowohl auf individueller als auch auf systemischer Ebene ansetzen. Am Beispiel der Adipositasprävention zeigt sich, dass Maßnahmen, die allein auf individuelle Verhaltensänderungen abzielen, oft unzureichend sind. Stattdessen sollten Ernährungsumgebungen sowohl durch regulatorische als auch verhaltensbezogene Maßnahmen gesundheitsförderlich gestaltet werden. BCI tragen dazu bei, das öffentliche Bewusstsein für den Zusammenhang zwischen Essverhalten und Umwelt zu schärfen.

Insgesamt bietet die Integration von BCI in Public-Health-Strategien die Möglichkeit, Verhältnis- und Verhaltensprävention zu verbinden und somit die Gesundheit der Bevölkerung nachhaltig zu verbessern.

## Abstract (en)

The application of behavioral and cultural insights (BCIs) in public health aims to enhance population health through a multidisciplinary and multimethodological approach. BCIs integrate findings from behavioral economics, psychology, and social and cultural sciences to address both individual behaviors and structural conditions.

Integrating BCIs into the public health action cycle (PHAC) allows for the consideration of behavioral, social, and cultural factors at every stage – from problem identification and strategy formulation to implementation and evaluation. Applying BCIs as a source to inform and extend the PHAC facilitates the development and implementation of more effective public health interventions that address both individual behaviors and systemic factors. In the area of obesity prevention, measures focusing solely on individual behavior change often prove insufficient. Instead, creating health-promoting food environments through regulatory and behavioral strategies is essential. BCIs can help raise public awareness about the link between eating habits and environmental factors.

Overall, integrating BCIs into public health strategies offers the potential to combine structural and behavioral prevention, thereby sustainably improving population health.

## Hintergrund

In den letzten Jahren haben sich durch Erkenntnisse der Verhaltenswissenschaft inspirierte politische Maßnahmen über den populären „Nudging-Ansatz“ [1] hinaus weiterentwickelt. Beim Nudging geht es ausschließlich darum, individuelles Verhalten in eine gewünschte Richtung zu lenken, ohne dabei bestehende Wahlmöglichkeiten einzuschränken. Behavioral and Cultural Insights (BCI) – also verhaltens-, sozial- und kulturwissenschaftliche Erkenntnisse – lassen sich hingegen auf vielfältigere Weise in der Gestaltung von Politik anwenden [2].

Im Bereich Public Health hat die 2020 gegründete „Behavioral and Cultural Insights Unit“ (BCI Unit) des Regionalbüros für Europa der Weltgesundheitsorganisation (WHO; [3]) maßgeblich zu diesem Emanzipationsprozess beigetragen. Aber auch das „EU Policy Lab“ der Europäischen Kommission empfiehlt in einer jüngsten Veröffentlichung ausdrücklich, verhaltenswissenschaftliche Erkenntnisse verstärkt für Veränderungen auf systemischer anstatt auf individueller Ebene zu nutzen [4]. Im Sinne einer „Behavioral Governance“, also einer auf verhaltenswissenschaftlichen Erkenntnissen basierten politischen Steuerung [5], können BCI in allen Phasen des Politikprozesses zum Einsatz kommen [6]. Dazu zählen die Problemdefinition, das Agenda-Setting, die Politikformulierung, die Implementierung und die Evaluierung. Anders als der in der Regel isoliert zur Anwendung kommende Nudging-Ansatz werden BCI in Kombination mit anderen Politikinstrumenten angewendet [7].

Vor dem Hintergrund verhaltenswissenschaftlicher Erkenntnisse – etwa der begrenzten Wirksamkeit von Nudging in Bezug auf ein Public-Health-Problem – kann die politische Entscheidung darin bestehen, die Lebensverhältnisse stärker zu regulieren und damit tiefer in die Entscheidungsfreiheit der Individuen einzugreifen [4]. Ein prominentes Beispiel ist die Einführung der abhängig vom Zuckergehalt von Getränken gestaffelten Zuckersteuer im Vereinigten Königreich im Jahr 2018, die zu einer signifikanten Reduktion des Zuckergehalts in Softdrinks führte [8]. BCI stellen demnach einen vielseitigen Ansatz zur Analyse und Bearbeitung von Public-Health-Problemen dar. Darüber hinaus können sie dazu beitragen, den bestehenden Dualismus zwischen verhaltensbezogener und verhältnisbezogener Gesundheitspolitik zu überwinden. Indem die Wechselwirkungen zwischen individuellem Verhalten und den ihnen zugrunde liegenden systemischen Strukturen beleuchtet und adressiert werden [9], bieten BCI einen Hebel zur Förderung der Bevölkerungsgesundheit.

Die BCI Unit der WHO weist darauf hin, das jedes Public-Health-Problem, wie z. B. Tabak- und Alkoholkonsum, eine verhaltensbezogene Komponente hat [3]. Verhaltens-, sozial- und kulturwissenschaftliche Erkenntnisse sind entscheidend, um die tieferliegenden strukturellen Ursachen von Public-Health-Problemen und deren verhaltensbezogenen Komponenten offenzulegen. Ein Beispiel hierfür ist die Erforschung der Gründe, warum der Prozentsatz adipöser Kinder und Jugendlicher zwischen verschiedenen Lebenswelten und sozialen Milieus variiert. So zeigt sich, dass Kinder und Jugendliche aus sozial benachteiligten Familien ein höheres Risiko für Übergewicht und Adipositas haben. Auf der Basis von BCI lassen sich informierte Entscheidungen hinsichtlich der Auswahl und des Designs von Public-Health-Interventionen treffen, um sowohl strukturelle Bedingungen als auch individuelles Verhalten gezielt zu beeinflussen.

Ausgehend von der Annahme eines bisher ungenutzten Potenzials von BCI für Public Health liegt dem Beitrag folgende Forschungsfrage zugrunde: Wie können verhaltens-, sozial- und kulturwissenschaftliche Erkenntnisse genutzt werden, um die systemischen Ursachen individuellen Gesundheitsverhaltens zu identifizieren und effektivere Public-Health-Interventionen zu entwickeln? Zur Beantwortung der Forschungsfrage erläutert der Beitrag zunächst die multidisziplinären und multimethodologischen Grundlagen von BCI. Im nächsten Schritt wird der Versuch unternommen, BCI in die 4 Phasen des Public Health Action Cycle zu integrieren, um den Fokus auf die Beeinflussung der systemischen Ursachen von individuellem Verhalten zu legen. Anschließend werden die Anwendungspotenziale eines durch die Nutzung von BCI ergänzten Public Health Action Cycle anhand des Beispiels der Adipositasprävention dargestellt. Der Beitrag endet mit einem kurzen Fazit.

## Multidisziplinär und multimethodologisch: BCI für Public Health

BCI steht für einen umfassenden Public-Health-Ansatz, der auf disziplinärem und methodologischem Pluralismus basiert. Demnach sollen Erkenntnisse aus dem gesamten Spektrum der Verhaltensforschung, insbesondere auch solche von sozialwissenschaftlichen Disziplinen wie Ethnografie, Humangeografie und Soziologie, für die Gestaltung, Implementation und Evaluation von Public-Health-Interventionen genutzt werden.

Ein Blick auf den Mainstream verhaltensbasierter Politik zeigt, dass bislang vor allem zwei Disziplinen prägend waren: die Verhaltensökonomie und die Psychologie [10]. Ihre Erkenntnisse zu Heuristiken und Verzerrungen (Bias), die rationale Entscheidungen verhindern, bilden den zentralen Bezugspunkt für verhaltenswissenschaftliche Politik. Diese Erkenntnisse basieren vorrangig auf randomisierten kontrollierten Studien und Laborexperimenten [11] und bilden die Grundlage für möglichst niedrigschwellige Interventionsformen. Nudging ist zu einem Synonym für zahlreiche verhaltenswissenschaftliche Techniken geworden. Ziel ist es, die sogenannte Entscheidungsarchitektur (Choice Architecture) durch soziale Normen, Standardeinstellungen (Defaults) und gezielte Informationshervorhebungen (Salience) zu beeinflussen [12]. Der gemeinsame Nenner dieser Formen der Verhaltensbeeinflussung ist die geringe Eingriffstiefe: Menschen werden möglichst „sanft“ in Richtung einer gewünschten Entscheidung gelenkt, wobei ihre individuelle Entscheidungsfreiheit gewahrt bleibt.

Ein viel diskutiertes Nudging-Beispiel aus dem Bereich Public Health ist die Gestaltung von Lebensmittelkennzeichnungen zur Förderung gesunder Ernährungsentscheidungen von Verbraucherinnen und Verbrauchern. Studien zeigen, dass Menschen ungesunde Lebensmittel kaufen, weil sie durch kognitive Verzerrungen wie die Attraktivität der Verpackung oder die Produktplatzierung im Supermarkt beeinflusst werden. Die Einführung von sogenannten Ampelkennzeichnungen wie dem „Nutri-Score“ [13], die Nährwertinformationen farblich codieren (grün für gesund, rot für ungesund), soll diesen Verzerrungen entgegenwirken und intuitive, gesündere Kaufentscheidungen fördern. Lebensmittelampeln verdeutlichen jedoch zugleich, warum eine disziplinär und methodisch eng gefasste Verhaltenspolitik zu unzureichenden Ergebnissen führt: Sie lässt soziale und kulturelle Rahmenbedingungen, in die das individuelle Konsumverhalten eingebettet ist, außer Acht.

Im Unterschied dazu erweitern BCI, die über die Methoden und Erkenntnisse der vermeintlichen Kerndisziplinen der Verhaltensforschung hinausgehen, den Fokus von Public-Health-Maßnahmen, indem sie die kulturellen, sozioökonomischen und sozialräumlichen Ursachen von Verhalten beleuchten und einbeziehen. Bei Lebensmittelkennzeichnungen stellt sich demnach die Frage: Wie müssen Ernährungsumgebungen gestaltet sein, damit Ampelsysteme als Teil eines Maßnahmenpakets wirksam sind (siehe Anwendungsfall Adipositas unten)? Der konzeptionelle Ausgangspunkt hierfür ist eine multidisziplinär und multimethodologisch ausgerichtete Verhaltensforschung [7]. Diese umfasst ein breites Repertoire an Methoden und Ansätzen, indem sie:

- qualitative Methoden wie Interviews und Fokusgruppen nutzt, um individuelle Einstellungen, kulturelle Werte und soziale Normen zu verstehen;
- ethnografische und partizipative Ansätze wie teilnehmende Beobachtungen und Prä-Post-Interventionen anwendet, um zu zeigen, wie soziale und räumliche Kontexte Verhalten von Menschen beeinflussen;
- mittels phänomenologischer Ansätze die tiefere Bedeutung von Alltagsverhalten und -praktiken untersucht, einschließlich kultureller und emotionaler Dimensionen, und
- mithilfe von quantitativen Methoden wie Umfragen und Metaanalysen Trends und Zusammenhänge zwischen sozioökonomischen Faktoren und Verhaltensgewohnheiten darlegt.

Führende Vertreter verhaltensökonomischer Forschung bzw. angewandter Verhaltenspolitik, wie Michael Hallsworth, Chief Behavioral Scientist des renommierten „Behavioural Science Team“ (BIT), haben den konzeptionell-methodologischen Weiterentwicklungsbedarf der Verhaltenswissenschaften erkannt. Mittlerweile scheint Einverständnis zu existieren, dass komplexe Probleme wie die Bewältigung der Klimakrise oder der Anstieg nichtübertragbarer Krankheiten durch eine Modifikation menschlicher Entscheidungsarchitekturen alleine nicht zu lösen sind [14]. Zwar gibt es Vorschläge zur Weiterentwicklung der Verhaltensforschung – etwa durch kontextsensiblere randomisierte kontrollierte Studien (RCTs) –, doch fehlt häufig die systematische Einbeziehung sozial- und kulturwissenschaftlicher Perspektiven [15]. Beispielsweise erweist sich das Nebeneinander soziologischer und verhaltensökonomischer Perspektiven und Ansätze in den Verhaltenswissenschaften [16] als äußert hartnäckig. Erst allmählich werden basale sozialwissenschaftliche Erkenntnisse, wie etwa die Dualität von Struktur und Handeln bzw. Handlungsfähigkeit (Human Agency; [17]), in den Mainstream der Verhaltensforschung integriert. Die Dualität von Struktur und Handeln beschreibt das Wechselspiel zwischen sozialen Strukturen – wie gesellschaftlichen Normen und Institutionen –, die das Verhalten von Individuen formen und prägen, und der Fähigkeit des Einzelnen, diese Strukturen durch eigenes Verhalten und Handeln aktiv zu beeinflussen und zu verändern.

Erst jüngst wurde in den Verhaltenswissenschaften auf die immense Bedeutung von Entscheidungsinfrastrukturen (Choice Infrastructures) für die de facto existierenden Entscheidungsoptionen von Menschen hingewiesen [18]. Demnach sind zum Beispiel in sozioökonomisch und sozialräumlich benachteiligten Wohngebieten individuelle Verhaltensmöglichkeiten, etwa was den Einkauf gesunder Lebensmittel angeht, strukturell eingeschränkt. In diesen sogenannten Food Deserts sind individuelle Verhaltensmöglichkeiten strukturell eingeschränkt. Hier ist Nudging, das individuelle Entscheidungsarchitekturen verändern will, etwa die Platzierung von Lebensmitteln in Supermärkten oder Essenangeboten in Schulkantinen [19], grundsätzlich ungeeignet für eine nachhaltige Verbesserung der öffentlichen Gesundheit.

Die renommierten Verhaltenswissenschaftler Chater und Loewenstein [20] sprechen sich deshalb für einen Paradigmenwechsel in der Verhaltenspolitik aus. Notwendig seien systemische Veränderungen (mittels „s-frame interventions“), um individuelle Entscheidungen (mittels „i-frame interventions“) in Richtung gesellschaftlich gewünschter Verhaltensweisen zu erleichtern. S‑frame-Interventionen beziehen sich auf systemische Veränderungen, die die äußeren Rahmenbedingungen und Strukturen beeinflussen, während I‑frame-Interventionen auf die direkten, individuellen Entscheidungsprozesse abzielen, um gewünschte Verhaltensweisen zu fördern. Demnach sind es nicht individuelle, sondern vor allem strukturelle, politische, ökonomische oder technologische Herausforderungen, die gesellschaftlichen Transformationszielen, wie der Etablierung einer „Planetary Health Diet“ [21], im Wege stehen. Eine multidisziplinäre und multimethodologische BCI-Forschung kann systemische Veränderungsbedarfe sichtbar machen. Sie kann zudem die Auswahl und Gestaltung von Public-Health-Interventionen gezielt unterstützen.

## Wie lassen sich BCI im Public Health Action Cycle nutzen?

Im Bereich Public Health stellt sich die Frage, wie sich ein umfassendes Verständnis von BCI – wie zuvor erläutert – theoretisch und praktisch nutzen lässt. Die Prämisse entsprechender Bestrebungen sollte es sein, verhaltenswissenschaftliche Erkenntnisse im Sinne der Verbesserung der Bevölkerungsgesundheit anzuwenden. Erreicht werden kann dieses Ziel durch die konzeptionelle Integration von BCI in den Public Health Action Cycle (PHAC; [22]). Der PHAC ist ein theoretisches Modell, das der Analyse von vier zentralen Phasen gesundheitspolitischer Interventionen dient: Problembestimmung, Strategieformulierung, Umsetzung und Bewertung [23]. Die vier Phasen des PHAC sind als idealtypisch zu verstehen, da sie in der Realität weder konsekutiv durchschritten werden noch zwangsläufig auftreten müssen [24]. Ungeachtet dessen hat sich der PHAC in der Public-Health-Forschung als Modell zur Analyse, Umsetzung, Weiterentwicklung und Evaluation von Politik zur öffentlichen Gesundheit bzw. Public-Health-Interventionen etabliert. Eine Integration von BCI in den PHAC hat somit das Potenzial, dessen Anwendungsmöglichkeiten noch einmal zu erweitern. Demnach lassen sich verhaltens-, sozial- und kulturwissenschaftliche Erkenntnisse in jeder Phase des PHAC nutzen:

1. *Problembestimmung*: BCI ermöglichen eine umfassendere Identifizierung der verhaltensbezogenen Ursachen von Public-Health-Problemen. Dabei kommen vor allem sozialwissenschaftliche (qualitative) Methoden wie Interviews, Fokusgruppen und teilnehmende Beobachtung zum Einsatz. Sie helfen, Einstellungen, kulturelle Werte und soziale Normen zu identifizieren, die gesundheitsrelevantem Verhalten in spezifischen sozialen und räumlichen Kontexten zugrunde liegen. BCI lassen sich in der Phase der Problembestimmung nutzen, um das Verhalten von (vulnerablen) Bevölkerungsgruppen zu erforschen. Ebenso ist es möglich, Einstellungen und Gewohnheiten von politisch und ökonomisch Handelnden in den Blick zu nehmen und zu fragen, inwiefern diese Public-Health-Reformen erschweren oder erleichtern [4].
2. *Strategieformulierung*: Die Kombination von klassischen Ansätzen der Verhaltensökonomie und Psychologie mit sozial- und kulturwissenschaftlichen Erkenntnissen erlaubt es, sowohl die Mikro- als auch die Makroebene von Public-Health-Problemen zu adressieren. Die Wechselwirkungen zwischen Lebensverhältnissen und individuellem oder kollektivem Verhalten machen eine Kombination aus regulatorischen und verhaltensbasierten Politikinstrumenten erforderlich. Der Fokus sollte dabei auf der Formulierung von *S‑frame-*Interventionen [20] liegen, die darauf abzielen, systemische Rahmenbedingungen dergestalt zu verändern, dass Menschen ein gesundheitsförderliches Verhalten dauerhaft erleichtert wird.
3. *Umsetzung*: Multidisziplinär entwickelte Public-Health-Strategien, die auf BCI basieren, müssen auf die jeweiligen Zielgruppen abgestimmt sein. Daher ist es wichtig, Zielgruppen frühzeitig einzubeziehen. So lassen sich mögliche Gegenreaktionen erkennen und geeignete Maßnahmen zur Prävention entwickeln. BCI geben Aufschluss über motivationale Treiber, aber auch habituelle Barrieren bei Individuen und Gruppen, die an der „Produktion“ von öffentlicher Gesundheit beteiligt werden sollen. Darüber hinaus ermöglichen BCI eine zielgruppengerechte Kommunikation. Das Ziel ist es, die Akzeptanz von Interventionen zu erhöhen. Gleichzeitig sollen ihre Wirkungen in unterschiedlichen Kontexten antizipiert und bei Bedarf angepasst werden. Ebenso sollten Public-Health-Expertinnen und -Experten sowie Multiplikatoren, wie zum Beispiel Angehörige von Gesundheitsberufen, partizipativ in der Umsetzungsphase beteiligt werden [9].
4. *Bewertung*: Die verhaltensbezogene Wirkung der Maßnahmen wird mittels Mixed-Methods-Ansätze umfassend evaluiert. Diese kombinieren quantitative Erhebungen (z. B. Umfragen, Metaanalysen) mit qualitativen Methoden (z. B. Interviews, teilnehmende Beobachtung). Dadurch lassen sich sowohl messbare Ergebnisse – inklusive unbeabsichtigter Effekte – als auch kontextbezogene Erkenntnisse erfassen. Das Ziel ist es, die (verhaltenswissenschaftliche) Evidenzbasis für zukünftige Interventionen zu stärken und damit die Handlungsoptionen von verantwortlichen Akteurinnen und Akteuren im Bereich Public Health zu erweitern.

Zusammenfassend bietet die systematische Integration verhaltens-, sozial- und kulturwissenschaftlicher Erkenntnisse und Methoden in den PHAC die Möglichkeit, die Interdependenzen zwischen gesellschaftlichen Verhältnissen und individuellem Verhalten präziser zu erfassen und zu analysieren. Für eine Public-Health-orientierte Politik eröffnet dies neue Handlungsmöglichkeiten – sowohl auf struktureller als auch auf individueller Ebene. Konkret können Maßnahmen entwickelt werden, die sowohl die Lebensverhältnisse als auch deren direkten Einfluss auf das Gesundheitsverhalten berücksichtigen. Wie dies im Bereich der Adipositasprävention umgesetzt werden kann, wird im Folgenden behandelt.

## Anwendungsfall: Gestaltung von Ernährungsumgebungen mithilfe von BCI

Chater und Loewenstein [20] ziehen eine ernüchternde Bilanz, was den bisherigen Beitrag der Verhaltenswissenschaften zur Eindämmung von Adipositas betrifft. Adipositas wird von den konventionellen Verhaltenswissenschaften zumeist als individuelles Problem verstanden. Ursache ist dabei der psychologische Effekt der Gegenwartsverzerrung (Present Bias), also die Tendenz zur unmittelbaren Bedürfnisbefriedigung beim Verzehr ungesunder Lebensmittel. Der Einfluss systemischer Faktoren auf Lebensmittelkonsum und Essverhalten wird hingegen kaum erkannt oder berücksichtigt. Dazu zählen etwa die Rolle der Nahrungsmittelindustrie sowie physische, wirtschaftliche, politische und soziokulturelle Kontexte, in denen Menschen mit dem Lebensmittelsystem interagieren. Anstatt die Bedeutung des individuellen Verhaltens und damit die persönliche Verantwortung für Adipositas zu betonen, sollten die Verhaltenswissenschaften konstruktiv zur Formulierung und erfolgreichen Umsetzung von systemverändernden Interventionen beitragen. Nur so könnten Menschen in die Lage versetzt werden, dauerhaft ihr Kauf- und Essverhalten zu verändern. Greift man Chaters und Loewensteins [20] Forderung auf, kann ein um BCI erweiterter PHAC einen wichtigen Beitrag zur Adipositasprävention leisten, indem er die systemischen Ursachen von vermeintlichen Verhaltensproblemen in Bezug auf Lebensmittelkonsum und Essverhalten aufzeigt und somit Public-Health-Interventionen zur Schaffung gesunder Ernährungsumgebungen unterstützt.

In den Gesundheitswissenschaften besteht weitgehender Konsens darüber, dass der sogenannte War on Obesity, also der Kampf gegen Adipositas, nicht allein mit Maßnahmen, die auf das individuelle Verhalten abzielen, gewonnen werden kann [25]. Die Mitverantwortung der Lebensmittelindustrie für das Essverhalten, insbesondere von Kindern und Jugendlichen, und damit auch für den Anstieg von Adipositas ist hinlänglich bekannt und beschrieben [26]. Ebenso sind die negativen Auswirkungen von sozialräumlichen Beschaffenheiten umfassend erforscht; in der Sozialgeografie werden solche Ernährungskontexte als „Foodscapes“ bezeichnet [27]. Ferner kann der Prozess der Akkulturation, verstanden als sukzessive Anpassung von Migrantinnen und Migranten an die mitunter ungesunde Esskultur des Aufnahmelandes, das Risiko, an Adipositas zu erkranken, erhöhen [28]. Derartige sozial- und kulturwissenschaftliche Erkenntnisse zu den strukturellen Rahmenbedingungen, die Lebensmittelkonsum und Essverhalten prägen, sollten bei der Bekämpfung von Adipositas systematisch berücksichtigt werden. Dass entsprechende Perspektiven durch die eingangs genannten Arbeitsgruppen der WHO und EU zunehmend in die Verhaltenswissenschaften integriert und aufgewertet werden, ist somit zu begrüßen [3, 4].

Verhaltensökonomische Maßnahmen zur Adipositasbekämpfung, wie das Platzieren von gesunden Lebensmitteln in Augenhöhe in Kantinen und Supermärkten oder gezielte Informationen von Zielgruppen über die sportliche Aktivität ihrer Peergroups, können das Kauf‑, Ess- und Bewegungsverhalten von Menschen positiv beeinflussen. In ungünstigen Ernährungsumgebungen, wie deprivierten Stadtteilen und Wohngegenden, in denen Übergewicht ein strukturelles Problem vulnerabler Gruppen darstellt, bleiben die genannten Interventionen jedoch oft wirkungslos. Ein möglicher Strategiewechsel in der Adipositasprävention könnte darin bestehen, das Scheitern verhaltensbasierter Interventionen als Beleg für die Notwendigkeit umfassender Verhältnisprävention zu nutzen. Im Kern würde dies eine stärkere Regulierung gesundheitsgefährdender Ernährungsumgebungen bedeuten. In Deutschland scheut die Politik bisher jedoch vor strikten Public-Health-Maßnahmen zurück – mit Ausnahme der weitgehenden Umsetzung des Rauchverbots im öffentlichen Raum. Weder die Einführung einer Zuckersteuer noch ein Werbeverbot für ungesunde Kinderlebensmittel werden durch eine politische Mehrheit unterstützt. Auch bei der Kennzeichnung von Lebensmitteln mit dem Nutri-Score setzt der Gesetzgeber bislang auf freiwillige Selbstverpflichtung der Lebensmittelindustrie – und nicht auf verpflichtende Vorgaben. Hierzulande gelten daher „Vorstellungen von einer Verhaltenslenkung des privaten Essverhaltens durch politische Regelsetzung … als weitgehend gescheitert“ [29]. Ungeachtet dieses Befunds bleibt es die zentrale Aufgabe der BCI-Forschung, auf die Dringlichkeit und grundsätzliche Zweckdienlichkeit einer strikteren Regulierung der Lebensmittelindustrie und der daraus resultierenden Effekte für die Beschaffenheit von Ernährungsumgebungen hinzuweisen.

Zielführend ist allerdings nicht alleine der Ruf nach Besteuerung und Verboten von bestimmten Lebensmitteln, sondern die Empfehlung unterschiedliche Politikinstrumente gezielt miteinander zu kombinieren, etwa um gesunde Lebensmittel finanziell attraktiver zu machen und den Konsum von ungesunden Produkten zu reduzieren. Ein Beispiel für die kombinierte Anwendung verschiedener Politikinstrumente zur Förderung gesunder Ernährung ist die gleichzeitige Senkung der Mehrwertsteuer auf Obst und Gemüse, die Einführung einer Herstellerabgabe auf zuckerhaltige Softdrinks sowie ein Werbeverbot für ungesunde Kinderlebensmittel [30]. Ein wesentlicher Beitrag der Forschung zu verhaltens-, sozial- und kulturwissenschaftlichen Erkenntnissen besteht somit darin, die wissenschaftliche Evidenz und das öffentliche Bewusstsein dafür zu stärken, dass sowohl das Essverhalten als auch der Lebensmittelkonsum in direktem Zusammenhang mit der regulatorischen Gestaltung von Ernährungsumgebungen stehen.

In der Umsetzung und Evaluation von Programmen zur Adipositasprävention ist es entscheidend, das Roll-out von entsprechenden Interventionen wissenschaftlich zu begleiten, eventuelle Barrieren für Verhaltensänderungen zu identifizieren sowie das angestrebte Zielverhalten fortwährend im Auge zu behalten [9]. BCI sind eine wichtige Ressource, um unterschiedliche Bevölkerungsgruppen für die Eindämmung von Adipositas zu sensibilisieren und an der Umsetzung entsprechender Maßnahmen zu beteiligen. Dafür ist es entscheidend, Motivationsfaktoren, Selbstwirksamkeit sowie soziale Normen und Werte zu analysieren. So lässt sich besser verstehen, welche Interventionselemente besonders wirksam sind – und welche weniger. Ein besseres Verständnis der genannten Faktoren ermöglicht eine kontextspezifischere Erfassung der strukturellen Bedingungen, die Adipositas begünstigen und Veränderungen im Lebensmittelkonsum sowie Essverhalten erschweren.

Des Weiteren können experimentelle Studien Aufschluss darüber geben, wie unterschiedliche Zielgruppen gegenüber einzelnen Komponenten von Präventionsprogrammen eingestellt sind, bevor diese umgesetzt werden. Hierzu zählt auch die Frage, ob und unter welchen Voraussetzungen Menschen zustimmend oder ablehnend auf regulative Eingriffe zur Stärkung der Verhältnisprävention reagieren [31]. Bisherigen Forschungsergebnissen zufolge hängt die Unterstützung von strikten Steuerungsmaßnahmen in erheblichem Maße von grundlegendem Vertrauen in die Politik, der Art der Informationsvermittlung (Framing) und der subjektiv wahrgenommenen Wirksamkeit der gewählten politischen Instrumente ab [32]. Derartige Erkenntnisse gilt es, gezielt in der öffentlichen Diskussion über das Für und Wider von regulativen Public-Health-Interventionen, wie der Einführung einer Zuckersteuer oder eines Werbeverbots für ungesunde Lebensmittel, zu kommunizieren.

Die genannten Ansätze verdeutlichen, wie die Verhältnisprävention im Bereich Adipositas durch den Einsatz von BCI gestärkt werden kann. Ein besseres Verständnis des Einflusses von Ernährungsumgebungen auf das Konsum- und Essverhalten ist entscheidend, um gesundheitsfördernde Lebensverhältnisse zu schaffen. BCI können den Entscheidungsprozess bei der Auswahl und Gestaltung von Interventionen zur Adipositasprävention gezielt unterstützen.

## Fazit

Verhaltens-, sozial- und kulturwissenschaftliche Erkenntnisse können dazu beitragen, Verhältnis- und Verhaltensprävention analytisch zu verbinden – zwei Bereiche, die bislang meist getrennt betrachtet wurden. BCI helfen dabei, den Zusammenhang zwischen Verhaltensreaktionen, wie impulsivem Essverhalten, und den Lebensweltbedingungen, wie etwa der hohen Dichte an Fast-Food-Restaurants, zu erklären und zu verstehen. Erkenntnisse aus einer multidisziplinären Verhaltensforschung sollten systematisch in den PHAC einfließen. Sie bieten wichtige Grundlagen für Analyse, Umsetzung, Weiterentwicklung und Evaluation gesundheitsbezogener Politik. Im Gegensatz zur Verhaltensökonomie und zu dem Konzept des Nudging, das auf moderate Anpassungen der individuellen Entscheidungsarchitektur setzt, können BCI ebenfalls regulative Eingriffe auf Systemebene rechtfertigen, um gesundheitsgefährdende Entscheidungsinfrastrukturen zu verändern. Am Beispiel der Adipositasprävention wird deutlich, dass die Förderung eines gesünderen Lebensmittelkonsums und Essverhaltens breiter Bevölkerungsgruppen maßgeblich von der politischen Regulierung der Ernährungsumgebung abhängt.
